# Supplementary figures and images for: Association between metabolic surgery and cardiovascular outcome in patients with hypertension: A nationwide matched cohort study
Source: PLoS Med. 2020 Sep 15;17(9):e1003307. doi: 10.1371/journal.pmed.1003307 (PMC7491727; doi:10.1371/journal.pmed.1003307)

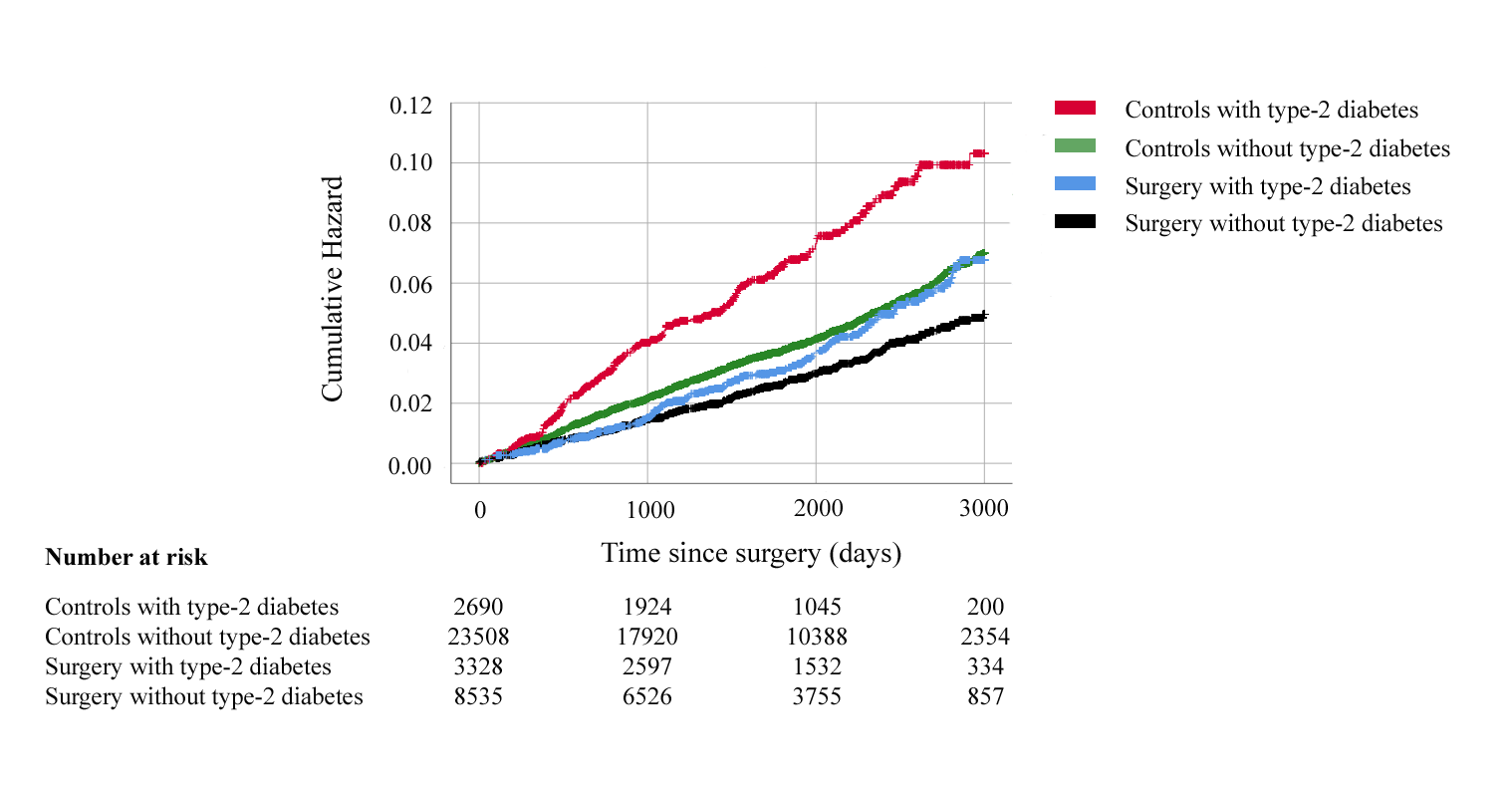

Supplement: S1 Fig — For study participants with T2DM, cumulative incidence at 3,000 days was 6.8% for the surgery group and 10.3% for the control group. For study participants without T2DM, cumulative incidence at 3,000 days was 5.0% for the surgery group and 7.0% for the control group. MACE, major adverse cardiovascular event; T2DM, type 2 diabetes. (TIF) [file pmed.1003307.s002.tif]

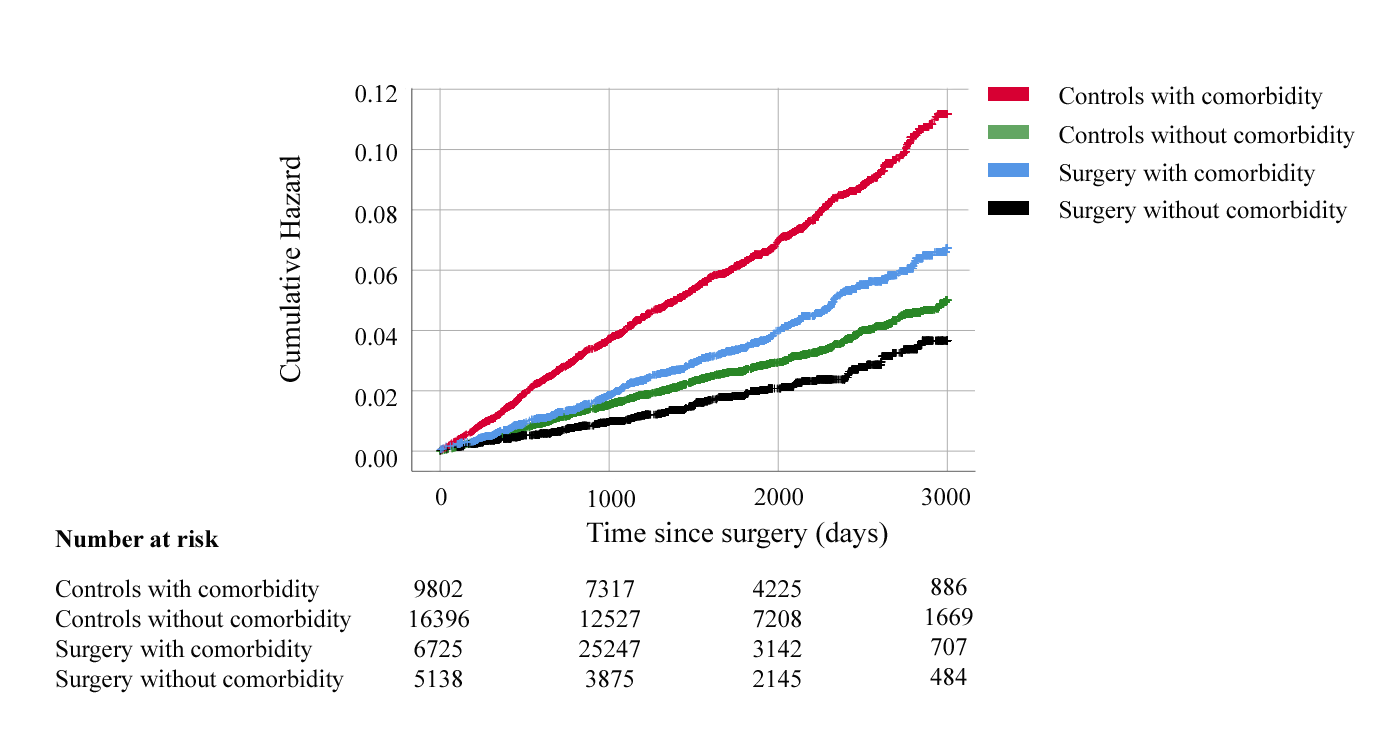

Supplement: S2 Fig — For study participants with comorbidity, cumulative incidence at 3,000 days was 6.7% for the surgery group and 11.2% for the control group. For study participants without comorbidity, cumulative incidence at 3,000 days was 3.7% for the surgery group and 5.0% for the control group. MACE, major adverse cardiovascular event. (TIF) [file pmed.1003307.s003.tif]

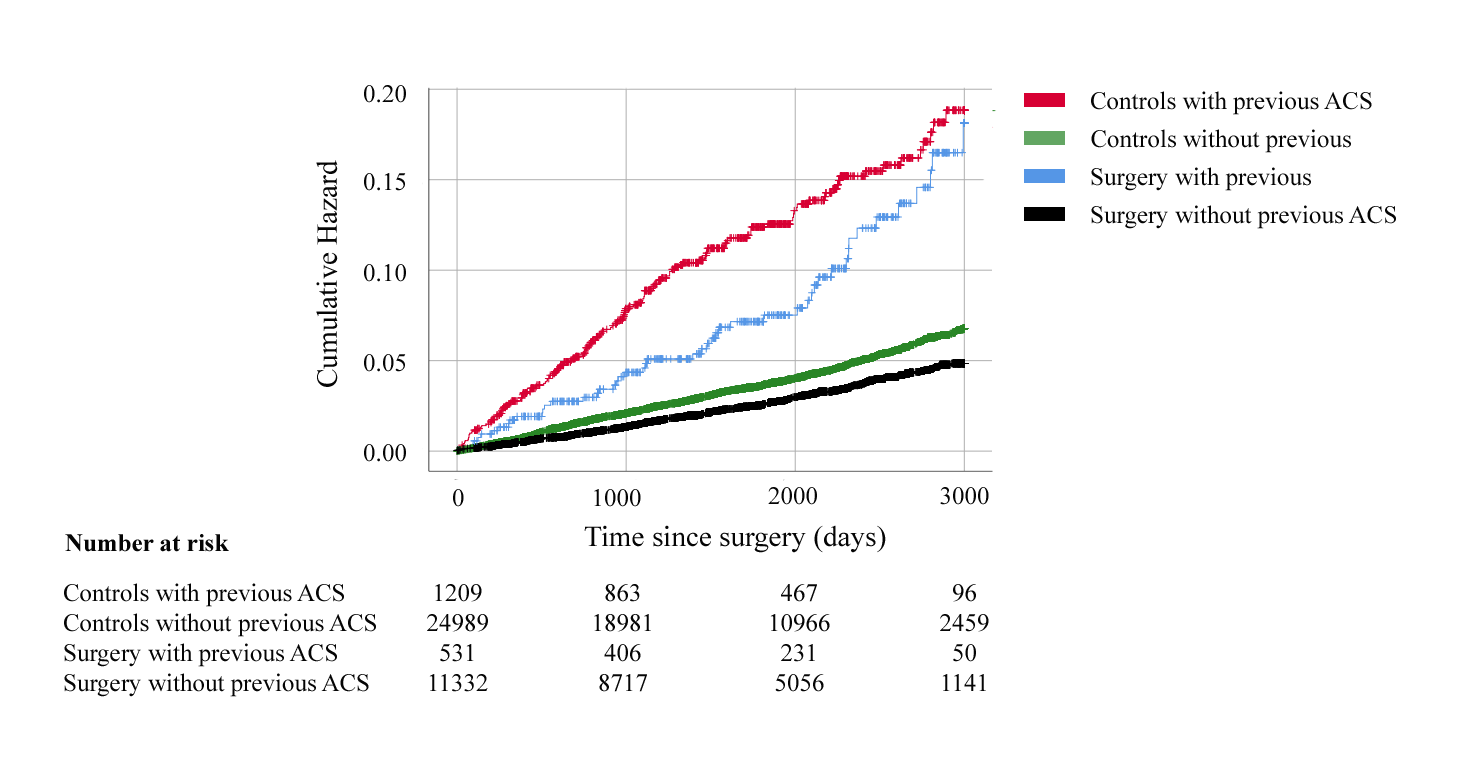

Supplement: S3 Fig — For study participants with previous ACS, cumulative incidence at 3,000 days was 18.1% for the surgery group and 18.8% for the control group. For study participants without previous ACS, cumulative incidence at 3,000 days was 4.8% for the surgery group and 6.8% for the control group. ACS, acute coronary syndrome; MACE, major adverse cardiovascular event. (TIF) [file pmed.1003307.s004.tif]
