# Supplementary material for: Association between metabolic surgery and cardiovascular outcome in patients with hypertension: A nationwide matched cohort study
Source: PLoS Med. 2020 Sep 15;17(9):e1003307. doi: 10.1371/journal.pmed.1003307 (PMC7491727; doi:10.1371/journal.pmed.1003307)
Supplement: Attachment — Submitted filename: response_hypertension.docx [file pmed.1003307.s005.docx]

Requests from the editors:

*In the last sentence of the Abstract Methods and Findings section, please briefly note some of the key limitation(s) of the study's methodology.

*Authors reply: The main limitations has now been added to the abstract. (Page 1, lines 57-58)*

*It would be good to clarify in the paper if the analyses reported here were planned out prospectively in a pre-existing protocol or analysis plan? Please state this (either way) early in the Methods section.

a) If a prospective analysis plan (from your funding proposal, IRB or other ethics committee submission, study protocol, or other planning document written before analyzing the data) was used in designing the study, please include the relevant prospectively written document with your revised manuscript as a Supporting Information file to be published alongside your study, and cite it in the Methods section. A legend for this file should be included at the end of your manuscript.

b) If no such document exists, please make sure that the Methods section transparently describes when analyses were planned, and when/why any data-driven changes to analyses took place.

c) In either case, changes in the analysis-- including those made in response to peer review comments-- should be identified as such in the Methods section of the paper, with rationale.

Authors reply: *No prospective protocol or analysis plan was documented or stored before the study began. However, such a plan was decided upon. Based on comments from reviewers, duration of hypertension was added to the analyses, and three categories for Education was used instead of four as in the original study plan. We also added stratified analyses for patients with T2D, any comorbidity, and previous acute coronary disease as supplementary analyses, and stratified analyses on MACE and remission of hypertension based on BMI. The methods section has been revised to provide a more transparent description of this. (methods section page 5-6, lines 150-154)*

*In the Methods section it would be good to add a note that the STROBE reporting guideline was used to guide reporting in the paper and there to call out the supporting information file that corresponds to the STROBE checklist.

*Authors reply: A reference to the checklist has been added to the revised manuscript. (Page 6, lines 154-155)*

-----------------------------------------------------------

Comments from the reviewers:

Reviewer #1: Association between metabolic surgery and cardiovascular outcome in patients with hypertension: a nationwide matched cohort study shows not only new, but very interesting data about a controversial issue in Metabolic Surgery, the impact on hypertension.

I have some comments and I hope they can improve your manuscript.

MAJOR

1.Lack of data on BMI is a critical issue about your manuscript. Revising other papers based on Sweden's registers, they showed the control group's BMI. Why do you not have it?

*Authors reply: We fully agree with the reviewer on this point. It has been very hard to construct control groups with BMI since it is not registered anywhere for the majority of the Swedish population. For certain subgroups of patients, mainly patients with diabetes, it is possible to find a suitable control group based on BMI. For patients with hypertension such a control group would either be based on a specific subgroup (such as diabetes), be based on data from military service (for all 18-year old men before the year 2000), or early in pregnancy for women. A few previous studies have used the diagnosis “E66.8” (obesity) to create a control group. However, this diagnosis is mainly used for patients considered for bariatric surgery, or a group of patients with superobesity and metabolic comorbidities. Hence, using such a control group would result in selection bias and by far overestimate treatment effects. We fully agree with the reviewer that this is a major limitation with the study and have presented it as such in the limitation section of the manuscript. Based on this comment and a comment from another reviewer, we have extended the discussion of this major limiation further. (Page 16, lines 380-382)*

2.Data about smoking would be very important. Do you have it?

*Authors reply: This is also a good point from the reviewer. No, we do not have reliable information on smoking for the control group. Based on this comment, we have added this as a limitation in the study as well. (Page 1, lines 57-58; Page 16, line 379)*

MINOR

1.In the abstract, the adjustment was made for more than one comorbidity. You should correct to comorbidities.

*Authors reply: The abstract has been revised accordingly. (Page 1, line 52)*

2.In methods: "The Scandinavian Obesity Surgery Register (SOReg) is a nationwide register for metabolic surgery" I suggest including Bariatric and Metabolic surgery.

*Authors reply: The methods section has been revised accordingly. (Page 5, line 129-130)*

3.When you cite ICD-10 and ATC-codes in the Definition of covariates, you should make clear from which coding system they are.

*Authors reply: We agree with the reviewer that the methods must be clearly and transparently presented and have changed the methods section in accordance with this comment. (several changes on Page 5-7)*

4.In the Outcome, do you think that it is necessary to include the ICD-10 codes? I suggest deleting them to make the text clearer.

*Authors reply: In our opinion it is necessary to present the outcome measures as clear as possible. However, most of these ICD-10 codes were presented earlier in the methods section and these have been removed from the outcome session. (Page 7)*

5.Table 1 should include the BMI.

*Authors reply: In the first version of the manuscript, we decided to present BMI in running text and not in the Table since BMI was available only for the intervention group. In response to this comment from the reviewer, the presentation has been moved to Table 1. (Page 9, Table 1)*

6.The follow-up time should be expressed in months. You can maintain days between parentheses.

*Authors reply: The presentation of follow-up time has been revised in accordance with this comment from the reviewer. (Page 9, line 240-241)*

7.Table 2 is a little confusing for me. Patients with T2DM submitted to Metabolic Surgery, for example, have more risk of MACE than control groups?

*Authors reply: Thank you for pointing this out! This is the multivariable analysis on risk for MACE presenting overall risk for MACE for the entire study group. Thus, metabolic surgery was a protective factor in this analysis, T2D was one of several risk factors. In response to this comment, we have revised the Table legend and we hope that this will clarify the presentation. In response to other reviewer comments, subgroup analyses for patients with comorbidities and T2D has been added as supplementary material which we hope will further clarify this. (Page 10, Table 2, and Supplementary figure 2)*

8.Although it is not the objective of your manuscript, can you show the other causes of mortality?

*Authors reply: While we agree with the reviewer that causes of mortality are highly interesting, the other causes of death lies beyond the scope of this manuscript. Besides cardiovascular comorbidity, the most common causes of mortality was cancer (24.3%), suicide/accidents (8.4%), and infectious disease (7.4%). A higher proportion of cancer as a cause was seen in the controlgroup, while suicides/accidents were overrepresented in the surgery group. We are a little concerned that presenting this data would divert focus from the outcomes of the present study. For this reason we would prefer not to present these data. If it remains a strong request from the Editor, and the reviewer then of course it can be included. (N/A)*

9.After the first appearance of type-1 and type-2 Diabetes Melitus you should use T1DM and T2DM that are frequently used in many manuscripts.

*Authors reply: The manuscript has been revised accordingly resulting in changes throughout the manuscript. (Changes throughout the manuscript)*

10.References: The 3-years results from GATEWAY were presented at AHA and the abstract is published, and you can include the results:

Schiavon CA, Bhatt DL, Santucci EV, Oliveira JD, Santos RN, Damiani LP, Machado RH, Noujaim PM, Halpern H, Monteiro FJ, Sousa MG, Amodeo C; Bortolotto L, Ikeoka DT, Cavalcanti AB, Berwanger O, Drager LF. Effects of Bariatric Surgery in Patients With Hypertension: 3-Year Outcomes From the Randomized GATEWAY Trial. Circulation. 2019;140:e972. DOI: 10.1161/CIR.0000000000000742.

*Authors reply: Thank you for sharing this highly interesting update from the very important GATEWAY-trial. Although the updated results from this trial are relevant to the discussion of our results both in terms of remission of hypertension and number of drugs, as we understand, the full manuscript has not been peer reviewed so far? If possible we would like to limit the references to those peer reviewed (N/A)*

Sincerely,

Carlos Aurelio Schiavon

-----------------------------------------------------------

Reviewer #2: Thank you very much for the opportunity to review this manuscript.

Overall, this is an interesting manuscript investigating a relevant topic for many patients, The paper is in line with many prior publications of the Scandinavian Obesity Surgery Register linked with national healthcare databases.

The manuscript is well-written, nicely prepared and clearly presented. It further supports the value of metabolic surgery in the treatment of a variety of comorbidties of metabolic diseases. However, there are some major issues with the underlying data (registry based and linking of many national databases using ICD-codes) which are acknowledged by the authors and can unfortunately not be addressed. Despite these limitations, I support the acceptance of the paper after revisions. The value of this paper is that it investigates for the first time patients specifically with hypertension independent of underlying comorbidities except obesity in a large cohort.

1. Also, if you have a relevant number of patients with a BMI <35kg/m2, could you provide the outcome stratified for these low-BMI patients? This questions would be of interesting to know and provide some answers if metabolic surgery is also of value in patients with BMI <35kg/m2. If you do not have these data, than you may be able to make an analysis comparing patients with BMI > and <40kg/m2. I do think that the notion that excess weight loss is associated with more hypertension remission should be related to with the preoperative BMI because EWL depends on the preoperative weight.

*Authors reply: Thank you for this comment! We agree with the reviewer that the question of lower BMI vs higher BMI are highly relevant. In response to this comment, we added post-hoc analyses for MACE and remission of hypertension. Since this also represented a change to the original study plan this was also noted in the methods section. (Page 10, lines 249-252, Page 11-12, lines 279-283)*

2. Could you provide a subgroup analysis for the following subgroups to better define which patients benefit as outlined below including figures like Fig. 2? It would be interesting to know whether one of the subgroup benefits in particular from metabolic surgery or if all benefit equally.

a. Patients with or without diabetes?

b. Patients with prior cardiac incident (prior MACE) c. Patients without any known comorbidities

*Authors reply: We have added the post-hoc subgroups analyses as supplementary files similar to Fig 2. These comparisons are not based fully on the matching procedure and should thus be viewed with caution. However, they may provide interesting results that could be the focus of future studies. (Supplementary Figure 2-4)*

-----------------------------------------------------------

Reviewer #3: I confine my remarks to statistical aspects of this paper. The general approach is fine but I have some issues to resolve before I can recommend publication.

p. 5 Why was a 1 to 10 matching chosen? How was the matching done? Much more detail is needed. (There are tons of ways to do matching)

*Authors reply: Thank you for this comment! The data set was based on a matched cohort of all patients operated with bariatric surgery in Sweden, matched 1:10 to the normal population (from a cohort of all Swedish citizens) using an exact match on age, sex and place of residence. Patients with hypertension and at least one matched control with hypertension were included in this study. Since we did not have appropriate numbers to estimate Power when the study was planned, a 1:10 match was decide on to reduce the risk for type-2 errors. (Page 5, line 134)*

p. 6 There is a typo People with 10 years of education don't fall in any category.

*Authors reply: Thank you for this comment! Education was defined as highest completed education. In Sweden, secondary education is currently 12 years of schooling, but historically 11 years. There has never been an option of 10 years of schooling. However, we fully agree with the reviewer that this is somewhat difficult for the reader to appreciate and we have therefore changed the definition of secondary education to 10-12 years. (Page 6, line 178)*

Why this division for education? In particular, why < 3 vs. > 3 years of higher ed? Are college degrees usually 3 years in Sweden? Why not degree vs. not?

*Authors reply: In the original study plan, higher education was divided into two categories. The rational for doing so was that “short” and “long” University degrees theoretically may represent somewhat different socioeconomic groups. However, in the present study no relevant difference was seen between these groups and in accordance with the comments from the reviewer we have merged this group into one (“Higher Education”). Since this represents a change to the original study plan, this has also been added to the methods section. (Page 5, lines 151-152; Page 6, line 179; Page 9, Table 1; Page 10, Table 2)*

p 10 Don't categorize years of hypertension. Categorizing continuous variables is nearly always a mistake. In *Regression Modeling Strategies* Frank Harrell lists 11 problems with this and sums up "nothing could be more disastsrous" Instead, leave years as years and use a spline to investigate nonlinearity

*Authors reply: The idea of including duration of hypertension was to separate short duration from established hypertension. This was not part of the original study plan, but was added after external input. While we agree with the reviewer that it would be much more informative to consider this variable as a continuous variable, we do not have data for the duration of time necessary to separate longer durations. For this reason, we separated the data for each year of duration up to 2 years. It is likely that longer duration is associated with lower chance of remission, but in our opinion this was the best possible way to use the data we have without adding additional bias to the study. In response to this comment from the reviewer we have included a test for linear trend, and a likelihood ratio for the two models, as well as added this problem to the limitations section. (Page 10, Table 2; Page 12, Table 3; Page 16-17, lines 390-394)*

The reference category for education needs to be specified.

*Authors reply: Thank you for noticing this! In response the reference category has been added to Table 2. (Page 10, Table 2)*

In the limitations, you should note the problems with BMI as a measure of obesity., See e.g. this blog post I wrote: <https://eur01.safelinks.protection.outlook.com/?url=https%3A%2F%2Fmedium.com%2Fpeter-flom-the-blog%2Fwhy-bmi-is-a-bad-measure-of-obesity-and-what-is-better-f8a62fc9ca49%3Fsource%3Dfriends_link%26sk%3D4b2ea559ab12853beb577764f83d151a&amp;data=02%7C01%7Cerik.naslund%40ki.se%7C2e86c88ad7194679017d08d808872c37%7Cbff7eef1cf4b4f32be3da1dda043c05d%7C0%7C0%7C637268723950000136&amp;sdata=T8O6%2BsPeJnYxQVBiK%2B9VYm0mho2iMLTB6q7hKn4UYbk%3D&amp;reserved=0>

*Authors reply: We agree with the reviewer that BMI has limitations. However, it is the definition used in studies on bariatric/metabolic surgery and also an important criteria for the indication of surgery. In response to this comment, we have included this in the limitations discussion. (Page 16, lines 378-380)*

Figure 3 Don't use stacked bar charts (see the work of William S. Cleveland). I think a mosaic plot might be good here, but it depends on what you are trying to show.

*Authors reply: Thank you for this input. The purpose of the stacked bars was to visualize changes in numbers of drugs before and after surgery. We used the stacked bars to allow comparisons with other clinical studies, in particular important studies on diabetes remission (Schauer et al NEJM 2017, doi:* [10.1056/NEJMc1703377](https://doi.org/10.1056/nejmc1703377) *), as well as the GATEWAY trial (doi:* 10.1161/CIR.0000000000000742)*. If possible we would prefer to keep the Stacked bar chart to allow straight forward comparison with the studies on diabetes medication after bariatric surgery, as well as the GATEWAY trial. If the Editor and reviewer persists on this request, then of course we will present this differently. To allow fair comparison to other studies we have added the proportions for each group in the figure. (Fig 3)*

Peter Flom
