# Supplementary material for: Association between metabolic surgery and cardiovascular outcome in patients with hypertension: A nationwide matched cohort study
Source: PLoS Med. 2020 Sep 15;17(9):e1003307. doi: 10.1371/journal.pmed.1003307 (PMC7491727; doi:10.1371/journal.pmed.1003307)
Supplement: Attachment — Submitted filename: rebutal_hypertoni2.docx [file pmed.1003307.s006.docx]

Requests from Editors:
Abstract
Background-please add a bit more context to illustrate the importance of this study
Please provide participant demographics
Please change “morbidly obese” to “patients with morbid obesity” in line with the principles of people first language. The same goes for “hypertensive” which should be revised to “with hypertension”

*Authors response: Thank you for these comments. We have revised the abstract accordingly (Page 1, lines 37, 45, 50-52; Page 2, lines 62 and 64)*

Author summary
Please change hypertensive to patients with hypertension
Line 90 probably doesn’t need a question mark

*Authors response: Thank you for these comments, we have revised the authors summary accordingly (Page 3, lines 86, 89 and 98)*


Comments from Reviewers:

Reviewer #1: Association between metabolic surgery and cardiovascular outcome in patients with
hypertension: a nationwide matched cohort study.
I would like to thank the authors for the responses and the improvement of the manuscript.
I still have some questions to you:

1. I understood that you do not have power to compare the two surgical techniques, but can you show the data about ACS events for each technique?

*Authors response: The study was not designed to compare the two surgical methods, and as previously stated, the study does not have enough Power to compare the methods. However, in response to this comment from the reviewer, data on the major outcome has been presented in the results section (Page 10, lines 245-247)*

2. I still have a problem with Table 2. You answered me that it is an analysis of the entire cohort, but the legend says matched cohort, what for me represents the control group. Please revise it.

*Authors reply: We agree with the reviewer that the results of each Table should be transparent and easily understood. We have revised the legend to make it more in line with other parts of the manuscript and also changed the language to people first language (Page 10, lines 254-256)*

3. The definition of remission of a comorbidity is a controlled disease without an active treatment. You did not show the data about blood pressure and, because of that, I am a little concerned about the remission rate based only in patients who were not taking medications. If you have the data about blood pressure, you should show it. If not, I think it is better to address this issue in the limitations.

*Authors reply: We do not have accurate data on blood pressure levels for the control group and have thus further specified this as a limitation with the study in accordance with the comment from the reviewer (Page 16, line 386)*

4. I still think that showing the others causes of mortality will enrich your paper, but I understood your point and I will let this decision to the Editor.

Authors response: *While we agree with the reviewer that causes of mortality are highly interesting* we are still *concerned that presenting this data would divert focus from the outcomes of the present study. For this reason, we would prefer not to present these data.*

5. Unfortunately, I did not find the supplement Figures in the manuscript.


Reviewer #2: Thank you for the revised manuscript. My comments and the other comments have been well addressed in my opinion.
However, there are 2 minor points:
1. I was not able to find the supplemental figures in the files
2. I think there is a labeling mistake in Fig. S4, second paragraph: I think it should be "without previous acute coronary syndrome" instead of type-2-diabetes.

*Authors response: Thank you for identifying this mistake, the legend has been updated accordingly (Page 21, Line 571)*


Reviewer #3: The authors have addressed my concerns and I now recommend publication

*Authors response: Thank you!*
